# Recommended number of strides for automatic assessment of gait symmetry and regularity in above-knee amputees by means of accelerometry and autocorrelation analysis

**DOI:** 10.1186/1743-0003-9-11

**Published:** 2012-02-08

**Authors:** Andrea Tura, Laura Rocchi, Michele Raggi, Andrea G Cutti, Lorenzo Chiari

**Affiliations:** 1 Institute of Biomedical Engineering, National Research Council, Corso Stati Uniti 4, 35127 Padova, Italy; 2 Department of Electronics, Computer Science and Systems, University of Bologna, Viale Risorgimento 2, 40136 Bologna, Italy; 3 INAIL Prostheses Centre, Via Rabuina 14, 40054 Budrio (BO), Italy

## Abstract

**Background:**

Symmetry and regularity of gait are essential outcomes of gait retraining programs, especially in lower-limb amputees. This study aims presenting an algorithm to automatically compute symmetry and regularity indices, and assessing the minimum number of strides for appropriate evaluation of gait symmetry and regularity through autocorrelation of acceleration signals.

**Methods:**

Ten transfemoral amputees (AMP) and ten control subjects (CTRL) were studied. Subjects wore an accelerometer and were asked to walk for 70 m at their natural speed (twice). Reference values of step and stride regularity indices (Ad1 and Ad2) were obtained by autocorrelation analysis of the vertical and antero-posterior acceleration signals, excluding initial and final strides. The Ad1 and Ad2 coefficients were then computed at different stages by analyzing increasing portions of the signals (considering both the signals cleaned by initial and final strides, and the whole signals). At each stage, the difference between Ad1 and Ad2 values and the corresponding reference values were compared with the minimum detectable difference, MDD, of the index. If that difference was less than MDD, it was assumed that the portion of signal used in the analysis was of sufficient length to allow reliable estimation of the autocorrelation coefficient.

**Results:**

All Ad1 and Ad2 indices were lower in AMP than in CTRL (P < 0.0001). Excluding initial and final strides from the analysis, the minimum number of strides needed for reliable computation of step symmetry and stride regularity was about 2.2 and 3.5, respectively. Analyzing the whole signals, the minimum number of strides increased to about 15 and 20, respectively.

**Conclusions:**

Without the need to identify and eliminate the phases of gait initiation and termination, twenty strides can provide a reasonable amount of information to reliably estimate gait regularity in transfemoral amputees.

## Background

Lower-limb amputees may present several gait deviations [1,2], with consequent increased energy cost, limited outdoor walking capacity [3] and a greater likelihood of developing muscle skeletal comorbidities [4]. In this view, several training sessions are necessary, before de-hospitalization of the patients [5-7]. However, what has been learnt during the rehabilitative/training sessions may be forgotten or wrongly recalled after some time when patients are back in their environment, and misuse of the prosthesis may occur again [3]. In our view, a "virtual gait trainer" would be very useful, i.e. an inexpensive system that the amputee can easily wear periodically to control, at home, the quality of gait in terms of symmetry and regularity and that can provide indications for improving the performances. In this context, gait symmetry is the degree of similarity of left and right steps, whereas gait regularity is the degree of similarity of consecutive strides. In fact, gait symmetry and regularity have been already analyzed in previous studies on subjects with lower-limb amputation [8,9].

The use of inertial sensors for analyzing gait has rapidly increased over the last few years. Recent advances in sensor manufacturing, computational power of portable systems, and wireless technology (ultimately translating into cheaper, smaller and less consuming sensors) have allowed extending the usage of inertial sensors (mainly accelerometers) beyond a merely clinical environment, to include also the home or outdoor environments, such as during activities of daily life or for long-term monitoring of signs and symptoms of specific pathologies [10-12].

In our view, key elements for designing such portable system are: 1) identification of relevant gait features and methods to measure them; 2) appropriate signal processing and feature selection; 3) evaluation of reliability and usability of the approach; 4) provision of practical guidelines for an evaluation protocol; 5) clinical validation of the evaluation protocol and of the rehabilitation outcomes. In a previous study [8] we faced the issues 1) and 2) of the aforementioned list, showing that the autocorrelation sequence of the acceleration signals measured on the thorax is appropriate to assess gait symmetry and regularity in transfemoral amputees and to provide a summary score to the user.

The present work moves from the outcomes of study [8] and aims to address items 3) and 4); in particular: i) presenting an algorithm to automatically and consistently compute symmetry and regularity indices (without the intervention of an expert operator); ii) assessing the minimum number of strides that are necessary for the appropriate assessment of gait symmetry and regularity through the autocorrelation sequence of the acceleration signals.

## Methods

### Participants

The subjects analyzed in this study are the same that were studied in [8]. Briefly, ten unilateral transfemoral amputees (AMP) wearing a lower-limb prosthesis with an electronically controlled knee (C-leg, Otto-Bock, D) were recruited at INAIL Prostheses Centre (Budrio, IT). All of them were confident walkers. Ten healthy subjects were also studied as a control group (CTRL). All participants were male and provided informed consent before data collection started. Main characteristics and walking parameters of the two groups of subjects are presented in Table 1.

**Table 1:** Main characteristics and walking parameters of the two groups

	*AMP*	*CTRL*	*P*
*Main characteristics *			

N	10	10	-

Age (years)	45.7 ± 3.1	27.7 ± 1.2	< 0.0001

Height (cm)	175.9 ± 1.7	179.8 ± 1.5	0.096

Body mass (Kg)^1^	75.8 ± 2.2	73.4 ± 3.1	0.48

Prosthesis use duration (months)^2^	127.2 ± 38.0	-	-

Electronic leg use duration (months)	37.9 ± 10.5	-	-

*General walking parameters*			

Natural walking speed (km/h)	4.0 ± 0.2	4.8 ± 0.3	0.036

Cadence_V _(stride/min)	51.23 ± 0.88	56.59 ± 1.75	0.061

Cadence_AP _(stride/min)	51.23 ± 0.88	56.57 ± 1.75	0.062

*Symmetry of step*			

Ad1_V _(unitless)	0.566 ± 0.036	0.939 ± 0.004	< 0.0001

Ad1_AP _(unitless)	0.359 ± 0.048	0.884 ± 0.007	< 0.0001

*Regularity of stride*			

Ad2_V _(unitless)	0.819 ± 0.016	0.930 ± 0.004	< 0.0001

Ad2_AP _(unitless)	0.763 ± 0.019	0.884 ± 0.008	< 0.0001

AMP - amputees; CTRL - control subjects. Subscript V - Vertical; AP - Antero-posterior. Of note, cadence from V and AP signals is virtually the same, as expected. Reported values are mean ± standard error (SE).

^1^with prosthesis in AMP

^2^from first fitting

### Equipment and experimental protocol

Acceleration signals were collected by the MEMS accelerometer of an XSens inertial measurement unit (MTx, Xsens Technologies B.V., NL), which has a full scale of ± 50 m/s^2^. The unit was placed on the thorax at the xiphoid process, following the guidelines from our previous study [8]. The sensitive axes of the accelerometer were manually aligned along the anatomical vertical (V), medio-lateral (ML), and antero-posterior (AP) axes. All the data were acquired at the sampling frequency of 100 Hz. The MTx unit applied an anti-aliasing hardware filter (1^st ^order, cut-off frequency = 28 Hz) before digitalising the acceleration signals. Data processing and analyses were performed in Matlab (The MathWorks Inc, US).

Subjects were asked to walk straight ahead along a hallway of the INAIL Centre, for a distance of 70 m. They were initially asked to walk at their natural speed. Subsequently, they were asked to walk slower than their natural speed for a second test and then faster for a third test. The order of the tests was fixed (natural, slow, fast speed). Each subject participated in two measurement sessions: after the first three tests, the operator removed the sensor from the thorax, and the subject was asked to rest for 15 minutes; then, a second operator placed the sensor on the thorax, and asked the subject to repeat the three gait tests in the same order. Thus, a total of 6 gait tests were acquired for each subject, two for each gait speed.

### Gait symmetry and regularity assessed by the autocorrelation sequence

The unbiased autocorrelation sequence of an acceleration signal *x(i) *can be computed by the following equation [13]:

(1) $$\text{Ad}\left(m\right)=\frac{1}{N-|m|}\overset{N-|m|}{\underset{i=1}{\sum }}x\left(i\right)\cdot x\left(i+m\right)$$

in which *N *is the total number of samples and *m *is the time lag expressed as number of samples. As shown in previous studies [8,13], when the autocorrelation of the acceleration signal is computed during gait, the first peak of Ad(*m*), Ad1, reflects the regularity of the acceleration between consecutive steps of the subject. This can be interpreted as a measure of the symmetry between steps performed by the prosthetic and the sound leg (or between left and right leg in CTRL). The second peak of Ad(*m*), Ad2, reflects the regularity of consecutive strides. Higher Ad1 (Ad2) values reflect higher step (stride) regularity (maximum possible value for Ad1 and Ad2 is 1). From the time lag between Ad1 and Ad2, given the sampling frequency, it is possible to compute the walking cadence (see Table 1).

Values of Ad1 computed from the acceleration signals along the vertical and antero-posterior axes will be indicated as Ad1_V _and Ad1_AP_, respectively. Similar nomenclature will be used for Ad2, i.e. Ad2_V _and Ad2_AP_. The medio-lateral acceleration signal, though available, was not analyzed in this study, since in our previous study [8] we found it poorly informative. Also, study [8] showed that gait tests at natural, slow and fast speeds provide essentially the same results. Thus, in this study we limited the analysis to the gait tests at the natural speed.

### Algorithm for the automatic identification of Ad1 and Ad2

**Figure 1:**
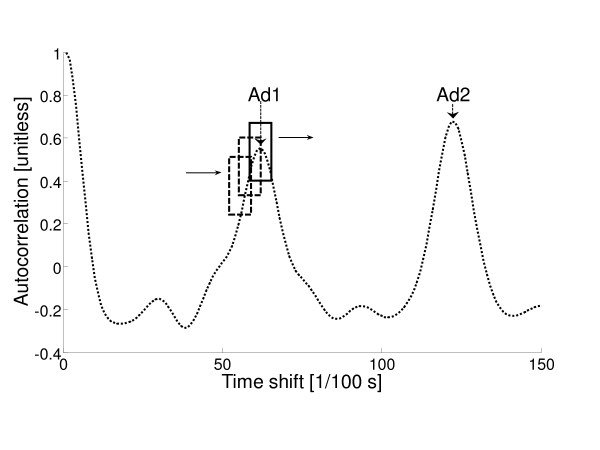
**Example of autocorrelation sequence with search window for maxima**. In this example, the autocorrelation sequence contains some spurious peaks. Ad1 and Ad2 peaks are indicated. The search window is shifted over the whole sequence (solid line: window in the position corresponding to Ad1 peak).

In this study we propose a new, simple algorithm capable of automatically identifying the peaks of the autocorrelation sequence corresponding to Ad1 and Ad2 coefficients. The algorithm operates as follows: *a) *A search window, three samples wide, is applied to the autocorrelation sequence. The window starts from the first sample of the sequence, and is shifted sample-by-sample until the sequence ends (Figure 1). *b) *In each position of the search window, the maximum value of the autocorrelation sequence is considered: if it is in the second of the three samples of the window, the value and related position in the autocorrelation sequence (time shift) are saved in a vector of maxima. *c) *When the search of maxima is finished, the vector of maxima is analyzed; since, by definition, Ad2 shows a time shift that is twice that of Ad1, the vector is searched to identify a couple of maxima whose time shifts are in a ratio of 2, with a given tolerance (10%). If one couple is found that satisfies this criterion, and both maxima are positive, that couple is assumed as a candidate for Ad1 and Ad2 (Ad1 being the maximum with lower time shift). *d) *Since in some cases such couple of Ad1 and Ad2 candidates may not be the actual Ad1 and Ad2, another possible couple of maxima is searched within the vector of maxima, but now limiting the search to those maxima whose time lag does not exceed twice the time lag of the identified Ad2 candidate (within the accepted tolerance). This time interval limitation is necessary to avoid the identification of different peaks in the autocorrelation sequence (such as Ad3 or Ad4, etc.). The limitation of twice the time lag of the first Ad2 candidate was empirically found adequate. *e) *If another couple of Ad1 and Ad2 candidates is identified, the new Ad2 candidate value is compared to the old Ad2 candidate value: if the former is greater, the new candidates are in fact assumed as being Ad1 and Ad2; otherwise, the old candidates are assumed as being Ad1 and Ad2.

**Figure 2:**
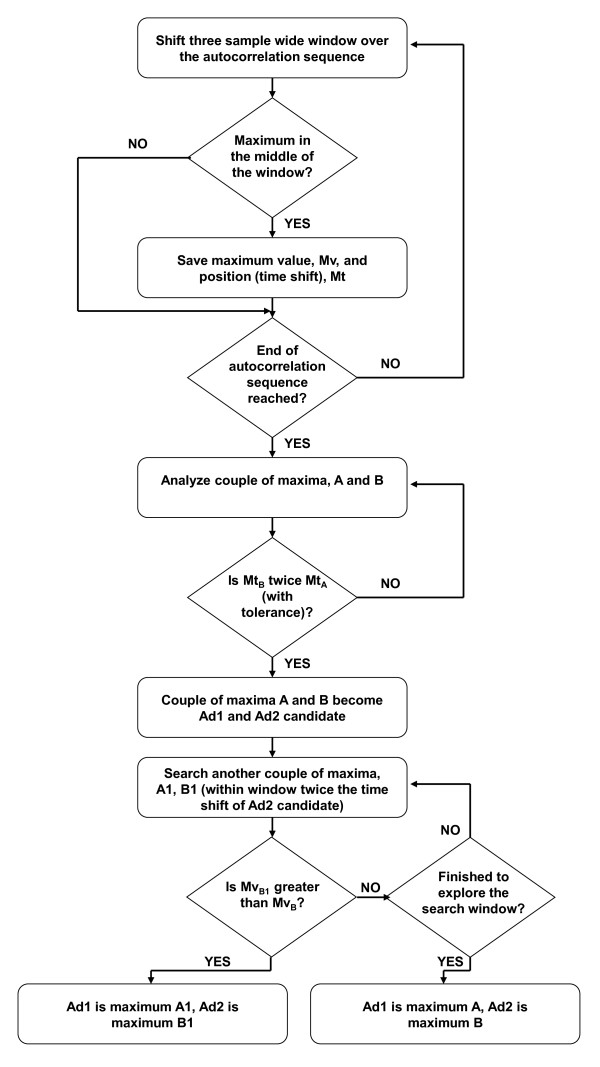
**Block diagram of the algorithm for automatic computation of Ad1 and Ad2**. Mv and Mt are the value and the time shift, respectively, of one maximum in the autocorrelation sequence. Maxima A and B are the first candidates to become Ad1 and Ad2. A1 and B1 are (possible) second candidates.

This algorithm was used for the computation of Ad1 and Ad2 in all the analyses previously described. A block diagram of the algorithm is depicted in Figure 2.

### Reference values for gait symmetry and regularity

For each of the gait tests, we initially considered only the central portion of the acceleration signals: we excluded from the analysis the first and last 1500 samples, so that we were highly confident that the transitional phases of gait initiation and termination were completely ignored (despite these exclusions the acceleration signals were still long enough: around 4500 samples on average). The obtained Ad1 and Ad2 coefficient values were assumed as the reference values (Ad1_ref_, Ad2_ref_) for gait symmetry and regularity estimation in each test.

### Gait symmetry and regularity from different portions of the acceleration signal

Firstly, we considered the central portion of the acceleration signals, as indicated above. For each signal, we performed the autocorrelation analysis for the computation of Ad1 and Ad2 coefficients beginning with a very small portion of the signal (100 samples). We then progressively enlarged the portion of the signal analyzed by steps of 100 samples. At each step of the procedure, Ad1 and Ad2 coefficients were calculated. Thus, for each acceleration signal we ended with a vector of Ad1 and Ad2 values that could be compared with the corresponding reference values previously obtained (see previous section: Ad1_ref_, Ad2_ref_).

Subsequently, we repeated the same procedure over the complete acceleration signals. Again, we obtained another (longer) vector of Ad1 and Ad2 values for each acceleration signal.

### Standard error of measurement and minimum detectable difference

As stated above, all subjects performed each gait test twice. This allowed us to calculate the standard error of measurement, SEM [14-16]. We analyzed the Ad1 and Ad2 reference coefficients, obtained from the central portion of the acceleration signals. For each coefficient (Ad1_V_, Ad1_AP_, Ad2_V_, Ad2_AP_), SEM calculation was performed separately for AMP and CTRL subjects.

SEM values were used to compute the minimum detectable difference, MDD, for each Ad1 and Ad2 coefficient. MDD indicates the minimum difference that must be observed between two measures of one variable to assume that the difference is "real", and not due to random errors, or systematic errors such as those, for instance, that are due to the incompetence of the operators (inter-rater variability). MDD was computed as $SEM\times 1.96\times \sqrt{2}$[16].

### Minimum number of strides for proper computation of Ad1 and Ad2 coefficients

For each gait test, we compared Ad1 and Ad2 obtained by a progressively larger portion of the acceleration signal with Ad1_ref _and Ad2_ref_. When the difference was lower than the corresponding MDD value, we assumed that the portion of the acceleration signal under analysis was sufficiently long to allow appropriate computation of the Ad coefficients. In fact, by definition, two measures of the same variable whose difference is less than the MDD have to be considered indistinguishable. From the calculated minimum length of the acceleration signal expressed in number of samples, with prior knowledge of the sampling frequency and the walking cadence of the subject (see Table 1), we were able to derive the minimum number of strides for appropriate Ad1 and Ad2 computation. Cadence of the subjects was computed from the reference portion of the acceleration signals.

### Statistical analyses

The possible inter-rater variability in the computation of Ad1_ref _and Ad2_ref _was assessed through Repeated Measures ANOVA. To this purpose, it was used a Matlab (The MathWorks Inc, US) function, called *rmaov1*, which was downloaded at the URL http://www.mathworks.com/matlabcentral/fileexchange (last checked: 14 July 2011).

Repeated Measures ANOVA was also used to assess possible differences between AMP and CTRL groups in the mean value of the Ad1 and Ad2 coefficients and the main characteristics of the subjects (see Table 1). Paired t-test was performed to assess differences between couples of variables in the same group of subjects. Linear regression analysis was also performed between some variables to investigate the possible effects of subject features (see Table 1) on our results. Before any test or analysis, each variable distribution was tested for normality, and logarithmic transformation was applied in the case of non-normal distribution. As regards Ad1 and Ad2 coefficients, given their peculiar type of distribution (defined only between 0 and 1) we systematically applied the Fisher's Z transformation. P values less than 0.05 were considered statistically significant. All the statistical analyses, except for the assessment of inter-rater variability, were performed with StatView (SAS Institute, Inc.).

## Results

AMP subjects were older than CTRL, but in any case they were middle-aged (Table 1). Height and body mass were not different in the two groups. The speed of natural walking was slightly higher in CTRL, but the cadence was not significantly different in the two groups.

As regards Ad1 and Ad2 coefficients, the reference values are reported in Table 1. Both Ad1 and Ad2 coefficients were higher in CTRL than in AMP, in agreement with previous findings [8].

Reported in Table 2 are the MDD of the Ad1 and Ad2 coefficients in the two groups: MDD were computed considering and excluding the systematic effects, which are predominately ascribable to the operators. Since the operator effect was not significant (see rater P-values), MDD values were virtually identical in the two cases.

**Table 2:** SEM and MDD of Ad1 and Ad2 coefficients

	*P* *rater*	*MDD random*	*MDD random+rater*
*AMP*			
Ad1_V_	0.36	0.065	0.065
Ad1_AP_	0.83	0.197	0.183
Ad2_V_	0.46	0.098	0.095
Ad2_AP_	0.77	0.095	0.090
*CTRL*			
Ad1_V_	0.29	0.023	0.023
Ad1_AP_	0.23	0.068	0.070
Ad2_V_	0.25	0.038	0.038
Ad2_AP_	0.73	0.099	0.094

AMP - amputees; CTRL - control subjects. MDD (unitless) are reported in the case of random error only, or with inter-rater effect also considered in the analysis. P values of the inter-rater effect are also reported.

The MDD values of the Ad1 and Ad2 coefficients made it possible to perform the subsequent analyses, aimed at identifying the minimum number of strides that are necessary to obtain coefficient values not distinguishable from the reference values (i.e., different from the reference values by less than the corresponding MDD values). For sake of brevity, we present results related to Ad1_AP _and Ad2_V _only (the coefficients that in a previous study resulted in being of major interest [8]).

**Figure 3:**
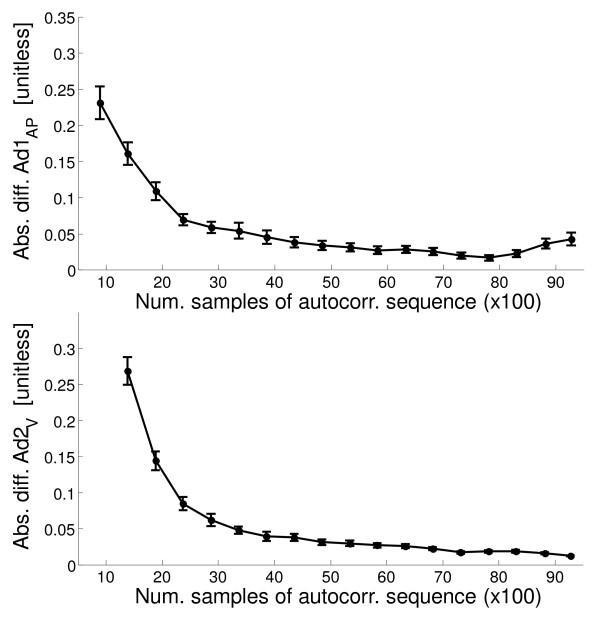
**Absolute difference of Ad1_AP _and Ad2_V _with their reference values in relation to the length of the acceleration signal portion**. Ad1_AP _- top; Ad2_V _- bottom. Data are reported as mean ± standard error (SE) (over all the subjects together). In these cases computations of Ad1_AP _and Ad2_V _are performed starting from the beginning of the acceleration signals.

Figure 3 (upper panel) shows the difference (absolute values) between Ad1_AP _and its reference value for increasing length of the acceleration signal portion considered. Similar information is reported for Ad2_V _(Figure 3, lower panel). Initially, the increment in the considered acceleration signal portion determines a strong decrease of the difference, whereas subsequently the difference is small or null: when the signal portion is sufficiently long for the analysis, longer segments become useless. In contrast, when the final portion of the signal is also included, the difference can worsen due to the negative effect of the last strides (just before stopping): this is clearly observable for Ad1_AP_.

Table 3 shows the minimum number of strides required for appropriate computation of Ad1_AP _and Ad2_V _(i.e., to get values not distinguishable from the reference values). When the acceleration signals were examined excluding the transitional phases of walking the number of strides to properly compute Ad1_AP _was very small, and not different between AMP and CTRL subjects. The minimum number of strides for appropriate computation of Ad2_V _was still small but greater than that for computation of Ad1_AP_, for both AMP and CTRL (see paired t-test, Table 3). When the acceleration signals were examined also including the initial and final portions, the minimum number of strides for appropriate computation of Ad1_AP _and Ad2_V _largely increased, due to the negative effect produced by the strides in the transitional phases. It should be noticed that in this case the minimum number of strides was significantly lower in AMP than in CTRL.

**Table 3:** Minimum number of strides for proper computation of Ad1_AP _and Ad2_V_

	*AMP*	*CTRL*	*P*
*Excluding initial and final 1500 samples in autocorrelation sequence*			

Min. number of strides (Ad1_AP_)	2.21 ± 0.23^1^	2.03 ± 0.21^2^	0.26

Min. number of strides (Ad2_V_)	3.57 ± 0.44^1^	3.43 ± 0.42^2^	0.49

*All samples in autocorrelation sequence*			

Min. number of strides (Ad1_AP_)	15.68 ± 2.00^3^	25.71 ± 1.85^3^	0.0014

Min. number of strides (Ad2_V_)	20.81 ± 1.76^4^	33.78 ± 1.99^4^	0.0033

AMP - amputees; CTRL - control subjects. Two cases were considered: acceleration signals analyzed excluding initial and final portions (upper part of the table), and also including those signal portions (lower part of the table). Reported values are mean ± standard error (SE).

^1 ^paired t-test: P = 0.0029; ^2 ^P = 0.0009; ^3 ^P = 0.0001; ^4 ^P = 0.0001

**Figure 4:**
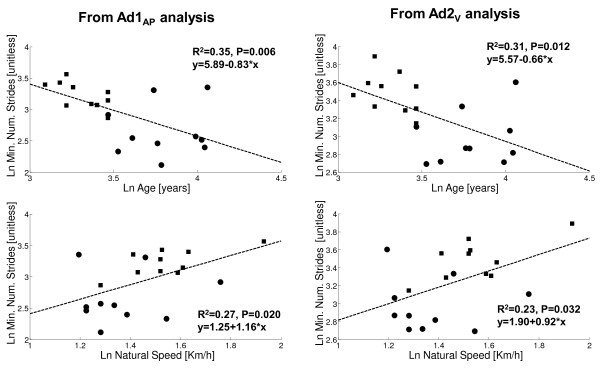
**Regression analysis between minimum number of strides and age or natural speed**. Circle - amputees; Square - control subjects. Left panel: minimum number of strides for Ad1_AP _computation (top: relationship with age; bottom: with natural walking speed). Right panel: minimum number of strides for Ad2_V _computation (top: relationship with age; bottom: with natural walking speed). R^2 ^and P values, and regression equations, are reported. Variable distributions are logarithmically transformed.

We also examined possible relationships between the minimum number of strides for appropriate Ad1_AP _and Ad2_V _computation and the main characteristics, as well as the general walking parameters, of the subjects considered all together (see Table 1). For this analysis we considered the minimum number of strides obtained from the whole acceleration signals (see Table 3). For both Ad1_AP _and Ad2_V_, a weak inverse relationship was found with age, and a weak direct relationship with the walking speed (Figure 4), but these weak relationships seem to be mainly due to the differences between AMP and CTRL rather than being intrinsic.

## Discussion

The main aim of this study was to address an issue that is crucial for the development of a system able to characterize and train gait for subjects wearing a lower-limb prosthesis, to be used out-of the hospital or rehabilitation center: the development of an automated and reliable algorithm for scores computation, and the calculation of the minimum number of strides that are necessary in these kinds of subjects for appropriate assessment of gait symmetry and regularity, through signals derived from inertial sensors and autocorrelation analysis. This would allow detecting differences in patients' performance over time, as well as between different prosthetic prescriptions and rehabilitation strategies.

From our previous study [8], Ad1_AP _and Ad2_V _were found being the autocorrelation coefficients of major interest (best ability to estimate step symmetry and stride regularity, respectively), hence in this study we focused the analysis on these two coefficients. It should be noted that in other studies, such as [13,17], the index considered for step symmetry was Ad1/Ad2, and not Ad1. In fact, if Ad2 is low, Ad1 might be low even if there is not an actual step asymmetry. For this reason, in some studies Ad1 was normalized by Ad2. However, in our study we preferred to be conservative, and hence we considered Ad1 without any normalization.

To evaluate the minimal portion of the signal that was necessary for reliable computation of Ad1_AP _and Ad2_V_, we had to define to what extent a possible difference with the Ad1_AP _and Ad2_V _reference values could be acceptable. To this aim, we referred to the computation of SEM and MDD [14-16].

As regards the analysis excluding initial and final signal portions, our results showed that the minimum number of strides for reliable computation of Ad1_AP _is very small, and not different between AMP and CTRL (slightly more than 2 strides in both groups, see Table 3). This means that when the gait is in a steady-state condition (no transitional phases), the autocorrelation-based method requires just a few gait cycles for the assessment of step symmetry, and the length of signal that is needed seems not to depend on the degree of symmetry that is expected for the population examined. Indeed, the request of the method of, on average, 2 strides of signal only (see Table 3) is near to what is minimally required by construction for the autocorrelation sequence computation. Similar considerations can be reported for Ad2_V_. The fact that for Ad2_V _the minimum number of strides is higher (between 3 and 4 strides on average) is due to the reason that the assessment of the stride quality obviously requires, by definition, more information than that required for the step. In fact, it is not surprising that the minimum number of strides for Ad2_V _is not far, on average, from being double than for Ad1_AP _(see Table 3).

As regards the analysis over the whole acceleration signal, without exclusion of the initial and final part of the gait, the minimum number of strides for both Ad1_AP _and Ad2_V _is, as expected, much higher than in the previous case. The transitional phase of the gait acts as a sort of noise in the coefficient computation, making it more difficult to obtain coefficient values similar to the reference values. In other words, several strides are necessary to make the deleterious effect of the first steps negligible. Again, the number of strides for Ad2_V _is larger than for Ad1_AP_, though the difference is less marked than in the case with no transitional phases. Furthermore, results show that the required number of strides for both Ad1_AP _and Ad2_V _is smaller in AMP than in CTRL. This is due to the fact that the requirements for reliability of coefficient computation are more severe for CTRL (i.e., lower MDD values: see Table 2), and hence the method, operating in a non-ideal condition (transitional phases included) has more difficulties in matching such requirements. However, in an outdoor environment, it would not be difficult to find paths of sufficient length: even with transitional phases of gait included, a relatively small number of strides are sufficient to make our approach working properly (see Table 3). On the other hand, it should be acknowledged that our results were obtained on a plain path. If the path is not perfectly plain (as it could be outdoor) the recommendations about the number of strides for reliable gait analysis may require further validation.

The statistical analysis performed to calculate SEM and MDD values also allowed us to assess the presence of possible inter-rater variability (every subject performed the gait test twice, with the help of a different operator in the two tests). We found that the two tests in each subject were not different, and hence there was no significant rater effect. Besides, this result also implicitly seems to suggest that test-retest variability, possibly due to fatigue effects, was not present as well.

Our results are hardly comparable with previous results. To our knowledge, no previous study addressed the problem of the minimum number of strides that are necessary for reliable estimation of the quality of gait in subjects with lower-limb prosthesis, not even with approaches different from those based on inertial sensors and autocorrelation analysis. However, we can compare our results with a couple of previous studies, though not performed on amputees. In [13], Moe-Nilssen *et al*. seem to claim that the number of steps for adequate gait assessment through autocorrelation sequence is around ten (i.e., five strides). This is in relatively good agreement with our results obtained in ideal conditions (no transitional phases), but not with those obtained in the non-ideal case. On the other hand, in another study [18] it was reported that the suggested length of a test for reliable assessment of gait regularity was 40 m. This is indeed more similar to the results that we found in this study.

## Conclusions

We addressed the problem of evaluating what the minimum number of strides is for reliable assessment of gait symmetry and regularity through accelerometry-based autocorrelation analysis in subjects with lower-limb prosthesis. We were able to reach the following conclusions and recommendations: when the gait includes transitional phases due to gait initiation, the number of strides to be performed over a rectilinear path should be around 15 for the assessment of step symmetry and 20 for stride regularity.

## List of abbreviations

Ad*i*: *i*-th peak of the autocorrelation sequence; ANOVA: Analysis of variance; AMP: Subject with amputation; AP: Antero-posterior axis; CTRL: Control subject; MDD: Minimum detectable difference; ML: Medio-lateral axis; Ref: Reference; V: Vertical axis; SEM: Standard error of measurement.

## Competing interests

The authors declare that they have no competing interests.

## Authors' contributions

AT has made substantial contributions to analysis and interpretation of data and has been involved in drafting the manuscript. LR has made substantial contributions to analysis and interpretation of data and has been involved in revising the manuscript. MR has made substantial contributions to acquisition, analysis and interpretation of data. AGC has made substantial contributions to conception and design, analysis and interpretation of data, and has been involved in revising the manuscript. LC has made substantial contributions to conception and design of the study and has been involved in revising the manuscript. All authors read and approved the final manuscript
